# Hyperthermia Enhances Efficacy of Chemotherapeutic Agents in Pancreatic Cancer Cell Lines

**DOI:** 10.3390/biom12050651

**Published:** 2022-04-29

**Authors:** Costanza E. Maurici, Robin Colenbier, Britta Wylleman, Luigi Brancato, Eke van Zwol, Johan Van den Bossche, Jean-Pierre Timmermans, Elisa Giovannetti, Marina G. M. C. Mori da Cunha, Johannes Bogers

**Affiliations:** 1 ElmediX NV, 2800 Mechelen, Belgium; costanza.maurici@elmedix.com (C.E.M.); britta.wylleman@gmail.com (B.W.); luigi.brancato@elmedix.com (L.B.); eke.vanzwol@elmedix.com (E.v.Z.); johan.vandenbossche@elmedix.com (J.V.d.B.); biamori@gmail.com (M.G.M.C.M.d.C.); 2 Laboratory of Cell Biology and Histology, Faculty of Medicine and Health Sciences, University of Antwerp, 2610 Antwerp, Belgium; robin.colenbier@uantwerpen.be (R.C.); jean-pierre.timmermans@uantwerpen.be (J.-P.T.); 3 Cancer Center Amsterdam, Department of Medical Oncology, Amsterdam UMC, 1006 Amsterdam, The Netherlands; elisa.giovannetti@gmail.com; 4 Cancer Pharmacology Lab, Fondazione Pisana per la Scienza, 56017 Pisa, Italy; 5 Applied Molecular Biology Research Group (AMBIOR), University of Antwerp, 2610 Antwerp, Belgium

**Keywords:** thermal therapy, cell proliferation, anticancer therapy, 5-fluorouracil, gemcitabine, cisplatin

## Abstract

Chemotherapy (CT) is the standard care for advanced pancreatic ductal adenocarcinoma (PDAC); however, with limited efficacy. Hyperthermia (HT) treatment has been suggested as a sensitizer to improve outcomes. However, the direct effect of the HT and CT combination is not fully understood. Therefore, we aim to assess the direct cytotoxic effect of HT in PDAC cells as monotherapy or in combination with chemotherapeutics. Different temperatures (37-, 40.5-, 41-, and 41.5 °C) and durations (6-, 12-, and 24 h) were tested in PDAC cell lines (BxPC-3, Capan-1, Capan-2, PANC-1, and MIA-PaCa-2). Different concentrations of gemcitabine, 5-fluorouracil, and cisplatin were also tested in these conditions. The impact on cell metabolic activity was determined by an MTS assay. Enhancement of chemosensitivity was assessed by a reduction in half-maximal inhibitory concentration ($IC_{50}$). HT and chemotherapeutics interactions were classified as antagonistic, additive, or synergistic using the combination index. HT inhibited cell proliferation in a cell type, temperature, and duration-dependent manner. The induction of apoptosis was seen after 6 h of HT treatment, eventually followed by secondary necrosis. The HT and CT combination led to an $IC_{50}$ reduction of the tested CT. At 12 h of HT, this effect was between 25 to 90% and reached a 95% reduction at 24 h. The additive or synergistic effect was demonstrated in all cell lines and chemotherapeutics, although, again, this depended on cell type, duration, and temperature. HT is cytotoxic and enhances the therapeutic effectiveness of gemcitabine, 5-fluorouracil, and cisplatin on PDAC cells. This result was further confirmed by the decrease in the expression of *RRM2*, *TS*, and *ERCC1* in BxPC-3 and Capan-2 cells. These observations warrant further study in specific subsets of PDAC patients to improve their clinical outcomes.

## 1. Introduction

Pancreatic ductal adenocarcinoma (PDAC) is one of the most aggressive solid malignancies and is characterized by poor prognosis, with a 5-year overall survival rate of around 10% [1]. For patients with locally advanced or metastatic cancer, as well as those with recurrence after surgery, the treatment of choice is chemotherapy. Most frequently used regimens include (combinations of) FOLFIRINOX (oxaliplatin, irinotecan, leucovorin, 5-fluorouracil), gemcitabine, nab-paclitaxel, and cisplatin [2,3], but their efficacy is low due to multiple factors. Poor drug penetration into the hypo-vascularized and the dense tumor stroma plays a key role in PDAC refractoriness to current therapies. This poor vascularization hampers oxygen delivery, resulting in a hypoxic microenvironment which further diminishes the sensitivity of cancer cells to chemotherapeutics [4] and irradiation [5,6].

Hyperthermia (HT) has been suggested as a chemosensitizer for improving drug distribution. Previous studies showed that heat increases vascular permeability and blood flow in the stroma, thereby enhancing oxidative stress [7] and the resorption of anticancer drugs into cancer cells [8,9,10]. Chemotherapy, thereby, becomes more effective without increasing toxic effects on the patient [10,11,12]. This has been demonstrated in many phase III clinical trials in patients affected by different tumor types [13,14,15,16,17].

HT can also induce selective tumor cell death when temperatures rise to the supraphysiological range (39–42 °C) [18]. This would be the result of several mechanisms, one of which is the increase in intracellular reactive oxygen species (ROS) [19,20,21,22,23]. Generally, at these temperatures, it is assumed that the main modality of cell death is apoptosis.

In addition to apoptosis induction, ROS are implicated to be involved in other types of cell death, such as autophagic cell death and necroptosis in cancer [7,23,24].

Aside from cellular ROS generation, there is some evidence that HT would also affect the cell’s ability to repair single-strand breaks [25]. Combined, HT would both induce DNA damage and impair DNA repair mechanisms.

Therapeutic modalities which rely on ROS, such as radiotherapy and some chemotherapeutic drugs, have already been demonstrated to benefit from HT [26]. Indeed, HT potentiates the efficacy of anti-tumor drugs by inhibiting tumor resistance genes such as *MDR1* gene expression and increasing the susceptibility of cells to several chemotherapy drugs [27].

Enhancement of the tumor-killing effect of chemotherapeutics by HT has been reported for several cancer types, using both in vitro and in vivo models [28,29,30,31]. Whole-body thermal treatment (WBTT) with gemcitabine and carboplatin emerged as a feasible treatment that led to some clinical benefit in a small cohort of PDAC patients. However, a parallel preclinical study showed no increase in the cytotoxicity of carboplatin and gemcitabine when HT was applied shortly (1 h) at 39–41.8 °C on the PDAC cell line DAN-G [32]. These controversial results and the urgent need for novel therapeutic strategies to overcome therapy resistance should prompt further studies to determine the optimal thermal dose for HT and chemotherapeutic combinations for PDAC.

Of note, HT can be applied as a local, regional, or whole-body treatment, and HT modalities vary from short-term (typically between 1 and 4 h) to long-term (exceeding 4 h). The thermotolerance of the liver and brain determines the maximal feasible target temperature, suited for clinical settings, to be equal to 41.8–42 °C, which may be maintained for several hours [33,34,35,36]. We have previously reported the safety and tolerability of WBTT at 41.5 °C for up to 3 consecutive exposures of 8 h in dogs and mini pigs [37,38].

In this study, we aimed to assess the direct cytotoxic effect of HT in PDAC either as monotherapy or in combination with chemotherapeutic drugs to identify an improved therapeutic modality for this type of cancer.

## 2. Materials and Methods

### 2.1. Cells and Reagents

Five different pancreatic cancer cell lines were used in this study: BxPC-3, Capan-1, Capan-2, MIA- PaCa-2, and PANC-1 (all from American Type Culture Collection, 20110 Manassas, VA, USA). These cell lines were isolated from human patients: the BxPC-3 from a pancreas adenocarcinoma; Capan-1 from a liver metastasis of pancreatic ductal adenocarcinoma; Capan-2 from a pancreatic adenocarcinoma tumor; MIA-PaCa-2 cells from an undifferentiated human pancreatic carcinoma; and PANC-1 from a carcinoma of the exocrine pancreas. Additionally, PWR-1E epithelial cells from a normal prostate were included as a healthy control.

Cells were cultured in an RPMI 1640 (BxPC-3, Capan-1 and Capan-2) or DMEM (MIA PaCa-2, PANC-1) medium containing, respectively, 15% (MIA-PaCa-2 and Capan-1) or 10% (other cells) of heat-inactivated Fetal Bovine Serum, supplemented with 2 mM L-glutamine at 37 °C in a 5% CO_2_ humidified atmosphere. For experimental use, the cells were detached from the culture flask and seeded at optimal seeding density (BxPC-3 at 5000 cells/well; Capan-1 at 7500 cells/well; Capan-2 at 10,000 cells/well; MIA-PaCa-2 at 1500 cells/well; PANC-1 at 5000 cells/well; and PWR-1E at 11,000 cells/well) in 96-well plates. All cells were kept at 37 °C in a humified atmosphere (5% CO_2_) for 24 h before hyperthermia treatment.

### 2.2. Chemotherapeutics and Hyperthermia Schedule

Three chemotherapeutic compounds were used: 5-fluorouracil, cisplatin, and gemcitabine (all from MilliporeSigma (owned by Merck KGaA) Fluery-les-Aubrais, France) diluted in DMSO (vehicle).

**Figure 1:**
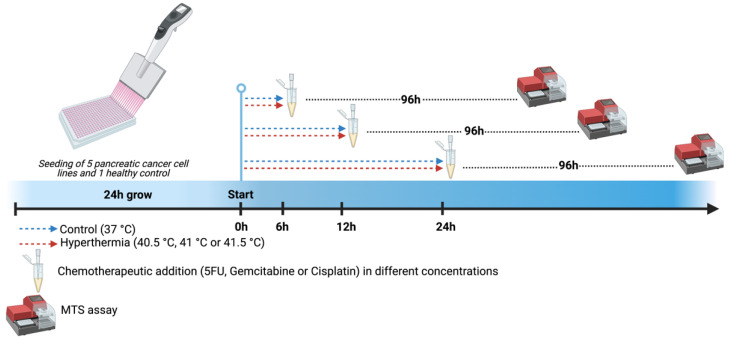
Experimental design. Pancreatic tumor cell lines (BxPC-3, Capan-1, Capan-2, PANC-1, and MIA-PaCa-2) were seeded and expanded for 24 h. Gemcitabine, 5-fluorouracil, and cisplatin were added immediately after hyperthermia treatment using different temperatures (40.5-, 41-, and 41.5 °C) and durations (6-, 12-, and 24 h). Controls were kept at 37 °C. After 96 h, the influence of chemotherapy and temperature on cell survival was determined by MTS assay. Created with BioRender.com.

The chemotherapeutic agents were serially diluted in an RPMI 1640 culture medium and tested in 9 therapeutically relevant tissue concentrations [39]. Vehicle controls were included in each condition. Prior to the evaluation at elevated temperatures, we performed an initial screening of the cytotoxic activity of the chemotherapeutic compounds at 37 °C (data not shown). To this end, we evaluated a series of concentrations of 2 to 5 doses below the observed IC50. This led to the final optimal concentrations, which can be seen in Supplementary Table S1. The experimental design is summarized in Figure 1. Briefly, the cell culture plates were incubated for different exposure times (6, 12, or 24 h) in a standard CO_2_ incubator (Binder CB160) preheated at different temperatures according to the experimental setup (37-, 40.5-, 41-, or 41.5 °C). Thereafter, the medium was replaced by a medium at 37 °C. The incubator was calibrated before starting the experiments using a calibrated independent probe.

Metabolic activity (MTS assay) was tested 96 h after exposure to the different temperatures. Each assay was performed in triplicate. These experiments were performed at Oncodesign (Dijon, France).

Additional experiments were performed to clarify the type of cell death induced by HT. First, we distinguished between apoptotic and necrotic cell death using an Apoptosis/Necrosis Detection Kit (ab176749, Abcam, Cambridge, UK) and by quantifying BAX/BCL-2 expression (RT-qPCR) during the various HT conditions. To further elucidate the potentiation of chemotherapy by HT, RT-qPCR data on differential expression of several genes associated with chemotherapy sensitivity were obtained. These experiments were performed at the Laboratory of Cell Biology and Histology, the University of Antwerp and the Medical Oncology Laboratory, Cancer Center Amsterdam, Amsterdam UMC.

### 2.3. MTS Assay

The in vitro cytotoxic activity was revealed by MTS assay using a tetrazolium compound (MTS,3-(4,5-dimethylthiazol-2-yl)-5-(3-carboxymethoxyphenyl)-2-(4-sulfophenyl)-2H-tetrazolium) and an electron coupling reagent (PMS, phenazine methosulfate). MTS is bioreduced by metabolically active cells into a formazan product that is directly soluble in a culture medium and is used as an indicator of cell viability, proliferation, and cytotoxicity.

At the end of the cell treatment, 40 µL of a 0.22 µM freshly filtered combined solution of MTS (20 mL at 2 mg/mL, Promega, Charbonnières-les-Bain, France) and PMS (1 mL at 0.92 mg/mL, MilliporeSigma (owned by Merck KGaA) Fluery-les-Aubrais, France) in Dulbecco’s Phosphate-Buffered Saline (DPBS, Cambrex, Paullo, Italy) was added to each well. Absorbance (Optical Density, OD) was measured at 492 nm in each well using an EnVision 2104 Multilabel Plate Reader (PerkinElmer, Villebon_sur-Yvette, France). For each point of measurement, the data were normalized to a control value at 37 °C and values were plotted in dose-response curves.

### 2.4. Quantitative Reverse-Transcriptase Polymerase-Chain-Reaction (RT-qPCR)

Total RNA of Capan-2 and BxPC-3 cells, subjected to 6, 12, and 24 h of HT at 41.5 °C and controls at 37 °C, was extracted using the TRIzol reagent (Invitrogen, Carlsbad, CA, USA), following the manufacturer’s instructions. To investigate apoptotic signaling, RT-qPCR was performed using TaqMan^®^ primers and probes for BAX and BCL2. Additionally, RT-qPCR was carried out for ribonucleotide reductase subunit 2 (*RRM2*), thymidylate synthase (*TS*), and excision repair cross-complementing-1 (*ERCC1*), respectively, as indicators for gemcitabine, 5-fluorouracil, and cisplatin sensitivity. The cDNA was amplified using the ABI-PRISM 7500 instrument (Applied Biosystems, Foster City, CA, USA) as previously described [40,41]. Gene expression values were normalized to Glyceraldehyde 3-phosphate dehydrogenase (GAPDH).

### 2.5. Apoptosis and Necrosis Assay

To further determine the type of cell death induced by HT, Capan-2 and BxPC-3 cells were labeled with an apoptosis/necrosis detection kit (ab176749, Abcam, Cambridge, UK). Cells were stained according to the manufacturers’ protocol immediately after removal from the hyperthermic conditions. This kit enables discrimination between healthy, apoptotic, and necrotic cells. Cells incubated at 55 °C for 1 h were used as a positive control for necrosis. Apoptosis was calculated relative to the respective cell lines growing at 37 °C, during the logarithmic growth phase. Images were acquired via Nikon Eclipse TI inverted microscope at a magnification of 4x. Quantitative analysis was performed using the Fiji/ImageJ software [42]. Microscopic evaluation was performed in sextuplicate.

### 2.6. IC50 and Combination Index Calculation

The data from in vitro experiments on pancreatic cell lines were analyzed in order to understand the impact of the combination of thermal treatment and chemotherapy medications on cell proliferation after different time periods and at given temperatures.

The combination index (CI) was used to determine the degree of drug and thermal treatment interaction as synergistic (CI < 0.9), additive (0.9 < CI > 1.1), or antagonistic (CI > 1.1).

The combination index concept was first introduced by Chou T.C. and Talay P. [43,44] to evaluate the degree of drug/drug interaction. Here, the original equation was adapted as follows:

(1) $$\mathrm{CI}=\frac{T_{\mathrm{HT}}-T_{\mathrm{ref}}}{{\mathrm{IT}}_{50,\mathrm{HT}}-T_{\mathrm{ref}}}+\frac{C_{50,\mathrm{drug}}}{{\mathrm{IC}}_{50,\mathrm{drug}}}={\mathrm{IT}}_{50,\mathrm{ratio},\mathrm{HT}}+{\;\mathrm{IC}}_{50,\mathrm{ratio},\mathrm{drug}}$$

where ${\mathrm{IT}}_{50,\mathrm{HT}}$ indicates the temperature required to inhibit cell viability by half of its maximal effect when only HT is applied to the cell lines, and ${\mathrm{IC}}_{50,\mathrm{drug}}$ is the half-maximal inhibitory concentration of a drug when chemotherapy is applied as monotherapy. $T_{\mathrm{HT}}$ represents the treatment temperature and $C_{50,\mathrm{drug}}$ the concentration required to provide a reduction in cell viability by half of its maximal effect when thermal treatment, at the temperature $T_{\mathrm{HT}}$, and drugs are combined. From now on, we will refer to $C_{50,\mathrm{drug}}$ as the combination dose for HT and chemotherapy.

$T_{\mathrm{ref}}=37\;{}^{\circ}C$ is subtracted from $T_{\mathrm{HT}}$ and ${\mathrm{IT}}_{50,\mathrm{HT}}$ to take into account the fact that the control temperature during the experiment is 37 °C. For the drug parameters $C_{50,\mathrm{drug}}$ and ${\mathrm{IC}}_{50,\mathrm{drug}}$ the control value is zero (i.e., no drug is present).

By rearranging the combination index equation, the dose reduction index (DRI) can be calculated for each drug in combination with HT. According to the definition by Chou et al. [43], the favorable DRI > 1.1 allows dose-reduction that leads to toxicity reduction in the therapeutic application. A 0.9 < DRI < 1.1 indicates no dose reduction while DRI < 0.9 indicates unfavorable dose reduction.

### 2.7. Statistical Analysis

${\mathrm{IC}}_{50,\mathrm{drug}}$ and $C_{50,\mathrm{drug}}$ were calculated by non-linear regression analysis using cell proliferation data on GraphPad Prism version 9.3.1 (GraphPad Software, La Jolla, CA, USA). ${\mathrm{IT}}_{50,\mathrm{HT}}$ was estimated with a data interpolation conducted with MATLAB using thin-plate spline (TPS). This method is ideal for examining the combined effect of two continuous predictors (i.e., time and temperature) on a single outcome (i.e., cell viability). Like other smoothing splines, TPSs are fitted using a generalized additive model (GAM), which does not require any a priori knowledge of the functional form of the data or the relationship of interest. Interpolation was performed since the twelve data points extracted from the in vitro experiments were not sufficient for the calculation of ${\mathrm{IT}}_{50,\mathrm{HT}}$ for all hyperthermia treatment conditions.

Two-way ANOVA followed by Dunnett’s multiple comparison test was used to assess the effect of HT on PDAC cell viability following exposure to different temperatures and durations compared to the control cell line at 37 °C. Tukey’s multiple comparison test was used to assess the effect of HT on the downregulation of *RRM2*, *TS*, and *ERCC1* and on the type of cell death in BxPC-3 and Capan-2 cells. The threshold for statistical significance was set at *p* < 0.05.

## 3. Results

### 3.1. Cytotoxic Effect of HT Is Cell Type-, Temperature-, and Time-Dependent

**Figure 2:**
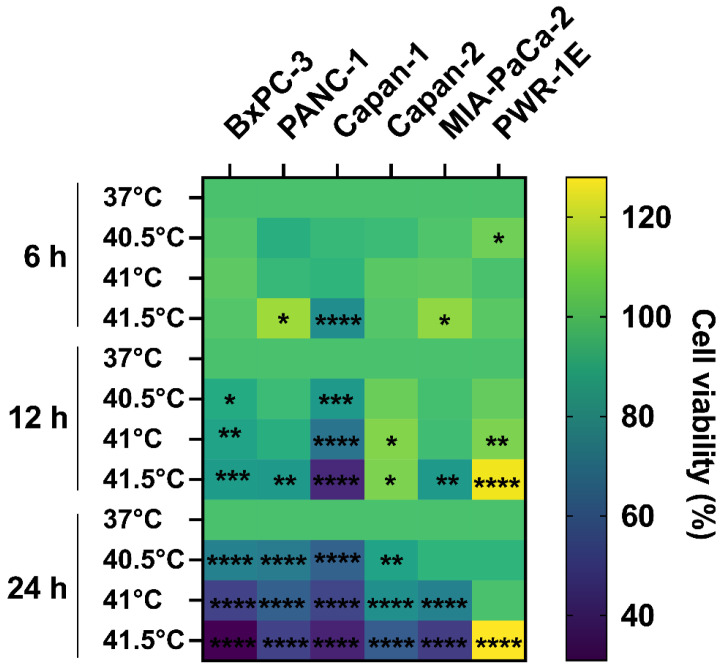
Effect of hyperthermia on pancreatic cancer cells. Heatmap showing the effect of hyperthermia on different cell lines for different durations (6, 12, and 24 h) and different temperatures (37 °C = control, 40.5-, 41-, and 41.5 °C). The color legend shows the percentage of relative cell survival compared to controls. There is a general trend of increased cell death with increased temperature and time of exposure. The healthy control cells are not killed by hyperthermia treatment. Results were expressed as relative cell viability as compared to 37 °C. Statistical significance * *p* < 0.05; ** *p* < 0.001; *** *p* < 0.0001; **** *p* < 0.00001.

To study the effects of HT alone on cell survival, treatments with different temperatures and durations were applied to untreated PDAC cells. HT reduced cell viability, as measured by MTS, to a different extent depending on the cell line (Figure 2). Capan-1 and BxPC-3 were the most thermosensitive and Capan-2 the most thermoresistant cell line. It was also observed that by increasing temperature and time of heat exposure, the viability decreased. HT did not appear to lead to a reduced viability of the control healthy cell line PWR-1E.

### 3.2. HT Induces Apoptosis of PDAC

After observing diminished cellular metabolic activity as measured by the MTS assay in several cell lines following HT, we sought to confirm whether this reflected the induction of cell death (e.g., apoptosis). Therefore, the most and least thermosensitive cell lines (e.g., BxPC-3 and Capan-2, respectively) were subjected to different durations of HT at 41.5 °C, followed by quantification of gene expression of the pro-apoptotic BAX and anti-apoptotic BCL-2 and evaluation of the externalization of phosphatidylserine (PS) on the outer membrane leaflet of the cells.

**Figure 3:**
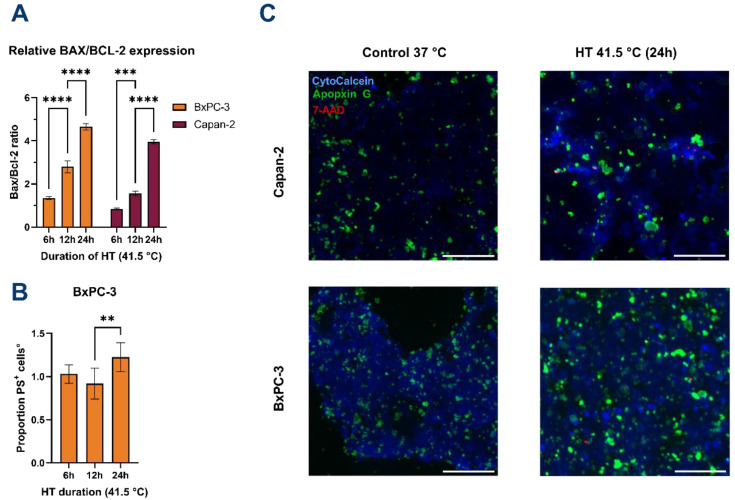
Hyperthermia induces apoptosis in PDAC cells. (**A**) BAX/BCL-2 expression ratio significantly increases over HT duration for both cell lines. Values were normalized for GAPDH expression and calculated relative to controls at 37 °C. (**B**) Expression of phosphatidylserine (PS) on the outer membrane is significantly increased after 24 h of HT only for BxPC-3. Data are normalized to controls at 37 °C. Data for Capan-2 are shown in Supplementary Figure S1. No significant differences in PS positivity were observed for Capan-2. (**C**) Representative fluorescence microscopy imaging of BxPC-3 and Capan-2 cells at 37 °C (control) and after 24 h at 41.5 °C. Viable cells are stained blue, apoptotic cells are stained green (PS), and necrotic nuclei are stained red. Images were acquired at 4× magnification. Scale bars represent 200 µm. All error bars represent 95% CI. Statistical significance ** *p* < 0.001; *** *p* < 0.0001; **** *p* < 0.00001.

With increasing HT duration, the BAX/BCL-2 ratio increases accordingly, resulting in a BAX/BCL-2 ratio of 1.35: 2.80: 4.65 for BxPC-3 after 6-, 12-, and 24 h, respectively (Figure 3A). Additionally, it appears that Capan-2 cells are affected distinctly, as after 6 h of HT a ratio of 0.84 is observed. However, after 12 and 24 h of HT, the ratio of BAX/BCL-2 increases to 1.56 and 3.95, suggesting that apoptosis is eventually induced in these cells as well.

No significant difference in PS presence was observed between exposure of 6 and 12 h for both cell lines. However, HT for 24 h significantly increased the number of PS positive cells for the BxPC-3 cell line (1.23-fold increase versus controls at 37 °C, Figure 3B). No significant increase was observed in PS positivity for the Capan-2 cell line. Necrosis was practically absent after 6 h of HT (0% and 0.8% for BxPC-3 and Capan-2, respectively).

Both cell lines showed an increase in necrotic cells following 24 h of HT (BxPC-3: 7% of the total number of cells; Capan-2: 8% of cells). HT at 55 °C for 1 h led to complete disruption of cellular integrity and over 99.99% reduction in viable cells for both cell lines.

These results show that exposure to long periods of HT induces mainly apoptotic cell death in at least BxPC-3 cells, demonstrated by the significant increase in cellular BAX/BCL-2 ratios and the PS presence in the outer membrane leaflet, eventually being followed by secondary necrosis.

Overall, a high presence of PS was detected on both BxPC-3 and Capan-2 cells at 37 °C (Figure 3C).

### 3.3. HT May Reduce the Required Dose of Chemotherapy

**Figure 4:**
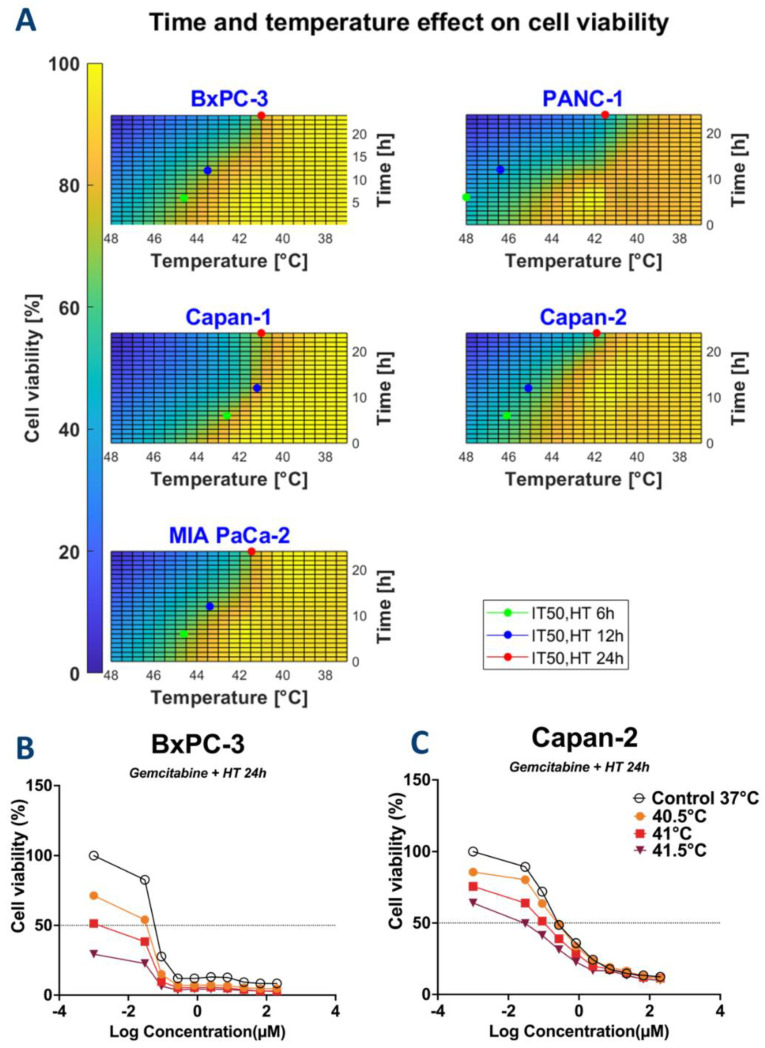
Combination of time and temperature effects on cell proliferation as illustrated by heatmaps for the calculation of ${\mathrm{IT}}_{50,\mathrm{HT}}$ and ${\mathrm{IC}}_{50,\mathrm{drug}}$ for thermal therapy (**A**) Heatmaps showing the relative cell viability in function of time and temperature were used to calculate $IT_{50,HT}$ for thermal therapy. ${\mathrm{IT}}_{50,\mathrm{HT}}$ values are shown for each of the cell lines at 6-, 12-, and 24 h. Heatmaps show that treatment duration increases the cytotoxic effect of thermal therapy observed by a lower ${\mathrm{IT}}_{50,\mathrm{HT}}$ (i.e., lower temperatures are needed to decrease cell viability by half of its maximal effect). (**B**,**C**): Dose curve response of BxPC-3 and Capan-2 to gemcitabine for 24 h.

${\mathrm{IT}}_{50,\mathrm{HT}}$ and ${\mathrm{IC}}_{50,\mathrm{drug}}$ data obtained from HT for 24 h and drugs administered as single therapy and combination doses between the two treatments are reported in Table 1. Values from all experimental conditions are reported in Supplementary Table S2. The calculated standard deviations for ${\mathrm{IC}}_{50}$ values were always found to be below 15% of the average. For clarity, these results are not added to Table 1. HT and CT induced dose-dependent cell killing of cultured PDAC cells. ${\mathrm{IT}}_{50,\mathrm{HT}}$ values were plotted in Figure 4A. Overall, by increasing the time of exposure, the required temperature to reach 50% of cell viability decreased. $IC_{50,drug}$ for each condition were derived from the dose curves, as reported in the representative Figure 4B,C and in the Supplementary Figures S2–S6.

**Figure 5:**
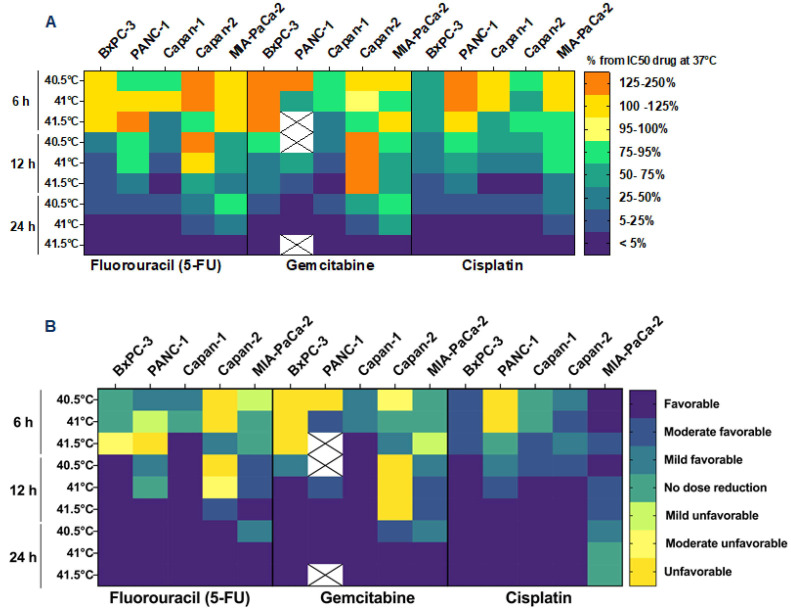
Hyperthermia enhances the cytotoxic effect of chemotherapeutic agents. Heatmap displaying the ${\mathrm{IC}}_{50,\mathrm{ratio},\;\mathrm{drug}}$ which represent the combined effect of hyperthermia and chemotherapy compared to single therapy (drug administered at 37 °C). Data are expressed as a percentage of ${\mathrm{IC}}_{50,\mathrm{drug}}$ at 37 °C experimental conditions. X indicates data not available (**A**). Dose reduction index values are plotted in (**B**) to further confirm the results expressed as relative ${\mathrm{IC}}_{50,\mathrm{ratio},\;\mathrm{drug}}$. Unfavorable: DRI < 0.8; Moderate unfavorable: 0.8 < DRI > 0.85; Mild unfavorable: 0.85 < DRI > 0.9; No dose reduction: 0.9 < DRI > 1.1; Mild Favorable: 1.1 < DRI > 1.3; Moderate favorable 1.3 < DRI > 1.8; Favorable DRI > 1.8. X indicates data not available.

The values ${\mathrm{IC}}_{50,\mathrm{ratio},\mathrm{drug}}$, defined as the ratio between the combination dose of chemotherapy and the ${\mathrm{IC}}_{50,\mathrm{drug}}\;\left(\mathrm{i.e.,}\;\frac{C_{50,\mathrm{drug}}}{{\mathrm{IC}}_{50,\mathrm{drug}}}\right),$ are reported in Supplementary Table S3 and plotted, as percentages, in Figure 5A. It was observed that the combination of the two treatments reduced the required dose of the chemotherapy compared to when it was administered as a single therapy. This effect starts at applications of 12 h, where a 50% reduction in drug concentration was observed and increased to a 75–95% reduction at 24 h of HT. Results were cell type-, temperature- and time-dependent, showing a clear trend of increased cytotoxic effects after 12 and 24 h exposure at the highest temperatures (41-, and 41.5 °C). This result is further confirmed by the calculation of the DRI (Figure 5B). As a general trend, after 12 and 24 h of thermal treatment, the combination of the two therapies is favorable and allows the reduction of the anticancer drug.

### 3.4. HT Has an Additive/Synergistic Anticancer Effect in Some Pancreatic Cancer Cell Lines

**Figure 6:**
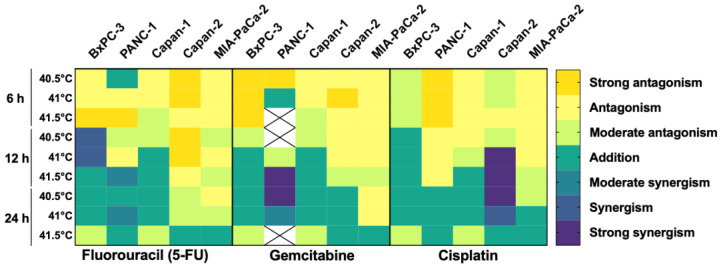
Hyperthermia and chemotherapeutics have additive/synergistic anticancer effects. Heatmap showing the combination index of hyperthermia and chemotherapy using different pancreatic cell lines and different temperatures and durations of hyperthermia. The combined effect between the two therapies is expressed as antagonistic, additive, or synergistic. Strong synergism: CI < 0.8; Synergism: 0.8 < CI > 0.85; Moderate synergism: 0.85 < CI > 0.9; Addition: 0.9 < CI > 1.1; Moderate antagonism: 1.1< CI >1.3; Antagonism 1.3< CI >1.8; Strong Antagonism CI > 1.9. X indicates data not available.

The combination index (CI) was calculated to determine whether the combination of HT and chemotherapy was synergistic, additive, or antagonistic. The results are reported in Supplementary Table S3 and summarized in Figure 6. Overall, HT enhanced the efficacy of gemcitabine, 5-fluorouracil and cisplatin in killing pancreatic cancer cells, with additive/synergistic interaction in most experimental conditions. However, this effect depends on multiple factors, specifically: time, temperature, drug, and cell type. Amongst the tested drugs combined with HT, 5-fluorouracil showed the highest synergy with BxPC-3, gemcitabine with PANC-1, and cisplatin with Capan-2 when HT was applied for 12 h. Of note, overall, HT enhanced the effect of chemotherapeutics, but it was difficult to see a synergistic effect when HT was applied for 24 h. We hypothesize that this limitation was due to the high effect already achieved by HT alone, as observed in other studies using high doses of chemotherapy [45].

### 3.5. HT Downregulates the Expression of Chemoresistance-Associated Genes

As we observed an overall additive or synergistic effect of HT on chemotherapeutic drugs, we aimed to investigate further the higher drug sensitization mechanisms of HT. More specifically, the downregulation of the expression of *RRM2* has been shown to increase PDAC sensitivity towards gemcitabine [46]. Additionally, low expression levels of *ERCC1* would increase sensitivity towards cisplatin [47,48]. Lastly, *TS*, a target of the (pro-)drug 5-FU, is upregulated in cancer cells as a mechanism for 5-FU resistance [49]. If HT increases the sensitivity of PDAC cells toward chemotherapeutic drugs, downregulation of these genes is expected.

**Figure 7:**
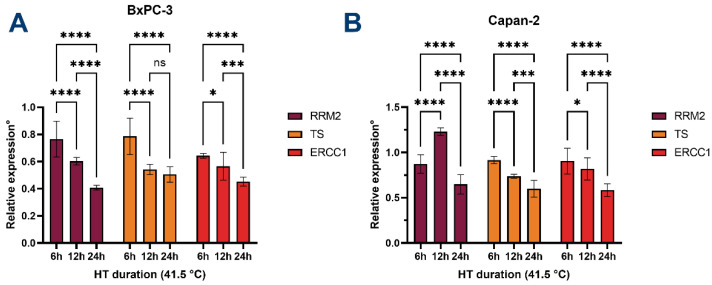
Hyperthermia leads to downregulation of RRM2, TS, and ERCC1. Relative expression of RRM1, TS, and ERCC1 in (**A**) BxPC-3 cells and (**B**) Capan-2 cells. Expression is normalized to GAPDH and calculated relative to cells incubated at 37 °C. Statistical significance ns = not significant; * *p* < 0.05; *** *p* < 0.0001; **** *p* < 0.00001.

Indeed, as depicted in Figure 7, HT induces significant decreases in the expression of *RRM2*, *TS*, and *ERCC1* in BxPC-3 and Capan-2 cells in all but two conditions compared to controls. The effect depends on the increasing duration of HT. Interestingly, 12 h of HT leads to a significant increase in the expression of *RRM2* in Capan-2 cells, which may be reflected in the observed antagonism with gemcitabine in these conditions. Furthermore, the expression of TS in BxPC-3 cells is downregulated after 6 h of HT and appears to plateau following durations exceeding 12 h. As such, these findings may partly explain why HT leads to higher sensitivity of PDAC cells to chemotherapy.

## 4. Discussion

In this study, we demonstrated that HT reduces the viability of pancreatic tumor cells and that its efficacy is cell type-, temperature-, and duration-dependent. Apoptosis was seen after 6 h of HT in at least BxPC-3 cells, demonstrated by the significant increase in cellular BAX/BCL-2 ratios and the PS presence in the outer membrane leaflet. Secondary necrosis was also observed. Capan-2 cells are differently affected as the ratio of BAX/BCL-2 only increases after 12 and 24 h of HT. Overall, a high presence of PS was detected on both BxPC3 and Capan-2 cells at 37 °C. This result is confirmed by a previous study [50] which reported that viable PDAC cells exhibit abnormally high levels of PS in the outer membrane leaflet.

Interestingly, the effect of HT on cell viability is strongly influenced by the cell type. We indeed used different PDAC cellular models that are representative of different PDAC characteristics, such as those possessing a more mesenchymal (PANC-1) or epithelial (BxPC-3) phenotype. Our results further proved the different thermal sensitivity of PDAC by confirming BxPC-3 as the most thermosensitive and Capan-2 as the most thermoresistant among the tested cell lines. Other research groups have also observed that some PDACs are inherently more resistant to drugs and hyperthermic effects [32].

In addition, we showed that HT potentiates the tumor-killing effects of gemcitabine, 5-fluorouracil, and cisplatin, which are used in chemotherapy regimens to treat PDAC patients [2,3]. One of the mechanisms that could be responsible for this observed effect is the downregulation of the expression of several genes associated with the mechanisms of action of the anticancer drugs (e.g., *RRM1*, *TS*, *ERCC1*). These effects are also dependent on the cell type, temperature, and duration of HT. These findings could support ongoing preclinical and clinical studies in the search for the optimal synergistic thermal and drug dose that could improve PDAC outcomes.

Despite many advancements in the current knowledge of PDAC genomics, most clinical efforts with experimental drugs have failed so far, and the prognosis for patients, unfortunately, remains poor. This grim prognosis is mainly due to its low treatment success rate. The tumor microenvironment, specifically the dense stroma, creates an immunosuppressive environment, blocking the efficacy of anti-cancer treatments [1]. HT has been proven to improve outcomes in the clinical setting [17], but the direct effect of the combination of hyperthermia and specific chemotherapeutics on the tumor cells remains unclear.

Previous work with a PDAC cell line [30] failed to show a cytotoxic effect of HT when applied as single therapy. However, the treatment was performed for only up to 90 min at 42 °C. In contrast, our results show that prolonged exposure to HT as monotherapy has a significantly stronger effect on cell viability, with the maximal effect potentially occurring after 24 h of exposure. These exposures are not easily attainable in clinical settings because this treatment is performed under general anesthesia. Nevertheless, a 6 h treatment was possible in a previous study [51] and is being applied in the ongoing ElmediX First-In-Human trial (NCT04467593, unpublished data). Notably, in this study, we show that exposure of PDAC cells of 6 h to HT induces a significant amount of apoptosis in PDAC cells. In addition, preliminary preclinical data suggest that the total HT duration may be delivered sequentially in 2–4 sessions with similar efficacy (data not shown). In other words, HT could potentially be fractionated in a similar fashion to radiotherapy, i.e., a total exposure time of 24 h could be achieved when divided over 4 sessions of 6 h.

Regarding the combination with chemotherapy, our results show that HT can lower the required doses of chemotherapeutics to achieve equal efficacy in vitro. This could improve outcomes and result in fewer side effects for the patients, considering that the current polychemotherapy (e.g., FOLFIRINOX) strategies provide only modest survival benefits, which are associated with high toxicity.

As discussed, several mechanisms may explain the potentiation of tumor-killing by heat treatment, some of which involve apoptosis induction by increasing intracellular ROS. Cancer cells are known to possess relatively high levels of base-line ROS, which promote tumorigenesis up to a certain threshold. However, ROS levels surpassing this threshold would be responsible for further increased protein misfolding, cellular stress, and the eventual initiation and maintenance of several pro-apoptotic signaling pathways [52]. High ROS levels have also been demonstrated to stimulate both the intrinsic and extrinsic apoptotic cell death pathways [52].

Extensive ROS signaling can lead to apoptosis via sustained P53 activation, resulting in the transcriptional downregulation of anti-apoptotic proteins of the Bcl-2 family [52]. HT also promotes apoptosis by upregulating BAX expression [27], a pro-apoptotic protein that can induce the release of cytochrome c, activate caspase proteases, and induce nuclear fragmentation.

Aside from cellular ROS generation, resulting in cell death, HT has also been shown to affect the DNA-repair mechanisms in cancer cells. For example, *BRCA2* levels in human cancer cells rapidly diminish during HT, resulting in impaired repair of double-strand DNA breaks [53]. Nevertheless, the exact mechanisms causing HT-induced apoptosis remain to be elucidated.

As previously reported [18,54], HT does not impair the cell viability of healthy cells. Heat shock proteins (HSP) have been suggested to play a role in the thermotolerance of the healthy cells [7,51]. Cancer cells have higher levels of partially activated HSP because they are coping with higher levels of constitutively misfolded proteins [55]. This is mainly due to the rapid rate of proliferation and hypoxia or acidic tumor microenvironment. So, a sufficiently increased level of ROS and misfolded proteins, induced by the combination of cytotoxic drugs and hyperthermia, may not be matched by the capacity of the intracellular HSP mechanism. Thus, the subsequent enhanced proteotoxic stress can be more toxic to cancer cells than to normal cells [56,57].

With our studies, we have been able to identify the optimal thermal dose for HT and chemotherapeutics combinations for different PDAC cells. However, the main limitation of this study is the fact that cells were evaluated in 2D culture conditions. Considering the characteristic tumor microenvironment of PDAC and the importance of 3D cellular interaction in proliferation and cell death processes, it is crucial that further research is performed, exploiting more complex in vitro and in vivo models. Especially considering that, for example, the diffusion of chemotherapeutic drugs can be significantly influenced in a 3D versus 2D cellular environment.

Although additional studies are needed for translation to the in vivo and the clinical situation, these findings do support further development of hyperthermal treatment for pancreatic cancer. Moreover, we hypothesize that HT could be a key modality for reducing tumor resistance since its efficacy is not limited by a low pO_2_ and low pH tumor microenvironment [34]. These conditions are typical for PDAC and constitute an important limiting factor for other anticancer treatments such as chemo- and radiotherapy [5,6,58,59]. However, the synergistic interaction of HT with specific anticancer drugs might overcome these challenges.

## 5. Conclusions

Hyperthermia is cytotoxic for pancreatic tumor cells, and its efficacy is cell type-, temperature-, and duration-dependent. As such, prolonged HT at 41.5 °C induces apoptosis rather than (primary) necrosis in PDAC cells. HT potentiates the tumor-killing effect of gemcitabine, 5-fluorouracil, and cisplatin and may have an additive or synergistic effect with chemotherapeutics drugs when used in combination with the optimal thermal and drug dose in specific PDAC patients. Ultimately, future work will help to better clarify the pharmacodynamics and biomarkers of what we hope will be a new therapeutic strategy in the fight against this therapy-resistant disease.

## Figures and Tables

**Table 1:** ${\mathrm{IC}}_{50,\mathrm{drug}}$, ${\mathrm{IT}}_{50,\mathrm{HT}}$ and combination doses between hyperthermia for 24 h and 5-fluorouracil, gemcitabine, and cisplatin.

			BxPC-3			PANC-1			Capan-1			Capan-2			MIA-PaCa-2		
**Drug**	**Time**	$T_{HT}\;({}^{\circ}C)$	$IT_{50,HT}\;({}^{\circ}C)$	$IC_{50,drug}\;(µM)$	$C_{50,drug}\;(µM)$	$IT_{50,HT}\;({}^{\circ}C)$	$IC_{50,drug}\;(µM)$	$C_{50,drug}\;(µM)$	$IT_{50,HT}\;({}^{\circ}C)$	$IC_{50,drug}\;(µM)$	$C_{50,drug}\;(µM)$	$IT_{50,HT}\;({}^{\circ}C)$	$IC_{50,drug}\;(µM)$	$C_{50,drug}\;(µM)$	$IT_{50,HT}\;({}^{\circ}C)$	$IC_{50,drug}\;(µM)$	$C_{50,drug}\;(µM)$
5-Fluorouracil	24 h	40.5	41	5.04	0.90	41.5	9.11	1.63	41	0.26	0.018	41.9	41.36	13.57	41.45	5.9	4.70
		41			0.04			0.12			0.0036			6.03			2.37
		41.5			0.00015			0.13			0.0002			1.18			0.03
Gemcitabine	24 h	40.5	41	0.06	0.01	41.5	19.34	0.20	41	0.06	0.0055	41.9	0.40	0.28	41.45	0.12	0.10
		41			0.0016			0.05			0.0013			0.08			0.06
		41.5			0.00006			-			0.0002			0.015			0.0008
Cisplatin	24 h	40.5	41	1.40	0.12	41.5	12.11	1.03	41	0.27	0.017	41.9	0.27	0.017	41.45	5.59	2.72
		41			0.0016			0.06			0.005			0.0053			0.33
		41.5			0.00012			0.014			0.0007			0.0007			0.0007

## Data Availability

Not applicable.

## Supplementary Materials

The following supporting information can be downloaded at: https://www.mdpi.com/article/10.3390/biom12050651/s1, Figure S1: Expression of phosphatidylserine (PS) on the outer membrane of Capan-2.; Figures S2–S6: Dose-response curve; Table S1: Serial dilution of chemotherapeutics; Table S2: ${\mathrm{IC}}_{50,\mathrm{drug}}$, ${\mathrm{IT}}_{50,\mathrm{HT}}$ and combination doses between hyperthermia and 5-fluorouracil, gemcitabine, and cisplatin (6-, 12-, 24 h); Table S3: ${\mathrm{IC}}_{50,\mathrm{ratio},\mathrm{drug}}$ (and combination index values of chemotherapy combined with hyperthermia treatment.
