# Supplementary material for: Hyperthermia Enhances Efficacy of Chemotherapeutic Agents in Pancreatic Cancer Cell Lines
Source: Biomolecules. 2022 Apr 29;12(5):651. doi: 10.3390/biom12050651 (PMC9138677; doi:10.3390/biom12050651)
Supplement: Supplementary file 1 [file biomolecules-12-00651-s001.zip › biomolecules-1624344-supplementary.pdf]

Supplementary Materials

Hyperthermia Enhances Efficacy of Chemotherapeutic Agents in Pancreatic Cancer Cell Lines

Costanza E. Maurici [1,†], Robin Colenbier [2,3,†], Britta Wylleman [1], Luigi Brancato [1], Eke van Zwol [1], Johan Van den Bossche [1], Jean-Pierre Timmermans [2], Elisa Giovannetti [3,4], Marina G. M. C. Mori da Cunha [1,†] and Johannes Bogers [1,2,5,*,†]

1 ElmediX NV, 2800 Mechelen, Belgium; costanza.maurici@elmedix.com (C.E.M.); britta.wylleman@gmail.com (B.W.); luigi.brancato@elmedix.com (L.B.); eke.vanzwol@elmedix.com (E.v.Z.); johan.vandenbossche@elmedix.com (J.V.d.B.); biamori@gmail.com (M.G.M.C.M.d.C.)
2 Laboratory of Cell Biology and Histology, Faculty of Medicine and Health Sciences University of Antwerp, 2610 Antwerp, Belgium; robin.colenbier@uantwerpen.be (R.C.); jean-pierre.timmermans@uantwerpen.be (J.-P.T.)
3 Cancer Center Amsterdam, Department of Medical Oncology, Amsterdam UMC, 1006 Amsterdam, The Netherlands; elisa.giovannetti@gmail.com
4 Cancer Pharmacology Lab, Fondazione Pisana per la Scienza, 56017 Pisa, Italy
5 Applied Molecular Biology Research Group (AMBIOR), University of Antwerp, 2610 Antwerp, Belgium
* Correspondence: john-paul.bogers@elmedix.com
† These authors contributed equally to this work.

Supplementary Tables

Supplementary Table S1. Serial dilution of chemotherapeutics applied in pancreatic cancer cell lines.

**5-Fluorouracil**	200 µM	66.6 µM	22.2 µM	7.4 µM	2.47 µM	0.82 µM	0.27 µM	0.091 µM	0.030 µM	Vehicle
**Gemcitabine**	200 µM	50 µM	12.5 µM	3.1 µM	0.78 µM	0.19 µM	0.049 µM	0.012 µM	0.0030 µM	Vehicle
**Cisplatin**	200 µM	66.6 µM	22.2 µM	7.4 µM	2.47 µM	0.82 µM	0.27 µM	0.091 µM	0.030 µM	Vehicle

Supplementary Table S2. $IC_{50,drug}$, $IT_{50,HT}$ and combination doses between hyperthermia and 5-fluorouracil, gemcitabine, and cisplatin. (6-, 12-, and 24 hours).

			BxPC-3			PANC-1			Capan-1			Capan-2			MIA-PaCa-2		
**Drug**	**Time**	T_{HT} (°C)	$IT_{50,HT}$ (°C)	$IC_{50,drug}$ (μM)	$C_{50,drug}$ (μM)	$IT_{50,HT}$ (°C)	$IC_{50,drug}$ (μM)	$C_{50,drug}$ (μM)	$IT_{50,HT}$ (°C)	$IC_{50,drug}$ (μM)	$C_{50,drug}$ (μM)	$IT_{50,HT}$ (°C)	$IC_{50,drug}$ (μM)	$C_{50,drug}$ (μM)	$IT_{50,HT}$ (°C)	$IC_{50,drug}$ (μM)	$C_{50,drug}$ (μM)
5-Fluorouracil	6 h	40.5	44.6	4.14	4.24	48	5.32	4.09	42.6	0.31	0.25	46.1	57.76	78.22	44.6	6.70	7.50
		41			4.38			5.91			0.32			96.02			7.31
		41.5			5.04			25.99			0.14			47.49			7.16
	12 h	40.5	43.5	4.38	1.25	46.4	7.69	6.39	41.2	0.35	0.13	45.1	60.83	79.21	43.4	7.06	5.26
		41			0.90			7.23			0.05			74.52			5.04
		41.5			1.03			2.98			0.0004			39.44			3.17
	24 h	40.5	41	5.04	0.90	41.5	9.11	1.63	41	0.26	0.018	41.9	41.36	13.57	41.45	5.9	4.70
		41			0.04			0.12			0.0036			6.03			2.37
		41.5			0.00015			0.13			0.0002			1.18			0.03
Gemcitabine	6 h	40.5	44.6	0.21	0.30	48	76.84	150.70	42.6	0.14	0.12	46.1	2.32	2.75	44.6	0.15	0.16
		41			0.29			56.14			0.11			2.27			0.14
		41.5			0.27			-			0.06			2.07			0.17
	12 h	40.5	43.5	0.08	0.06	46.4	25.02	-	41.2	0.07	0.02	45.1	3.06	5.40	43.4	0.13	0.10
		41			0.024			17.57			0.009			5.44			0.09
		41.5			0.022			5.57			0.00005			3.93			0.08
	24 h	40.5	41	0.06	0.01	41.5	19.34	0.20	41	0.06	0.0055	41.9	0.40	0.28	41.45	0.12	0.10
		41			0.0016			0.05			0.0013			0.08			0.06
		41.5			0.00006			-			0.0002			0.015			0.0008
Cisplatin	6 h	40.5	44.6	2.39	1.77	48	5.14	7.31	42.6	0.32	0.33	46.1	13.55	10.52	44.6	6.30	6.43
		41			1.75			7.63			0.34			9.76			6.40
		41.5			1.69			5.32			0.18			11.58			5.54
	12 h	40.5	43.5	1.56	0.67	46.4	5.27	4.07	41.2	0.28	0.21	45.1	0.28	0.21	43.4	4.67	4.35
		41			0.57			3.25			0.07			0.07			3.56
		41.5			0.36			2.18			0.0018			0.0019			2.17
	24 h	40.5	41	1.40	0.12	41.5	12.11	1.03	41	0.27	0.017	41.9	0.27	0.017	41.45	5.59	2.72
		41			0.0016			0.06			0.005			0.0053			0.33
		41.5			0.00012			0.014			0.0007			0.0007			0.0007

Supplementary Table S3. $IC_{50,ratio,drug}$ and combination index (CI) values of chemotherapy combined with hyperthermia treatment. CI) lower than 0.9 indicates synergism, between 0.9 and 1.1, additivity and higher than 1.1 indicates antagonism.

		BxPC-3			PANC-1			Capan-1			Capan-2			MIA-PaCa-2		
**Drug**	**Thermal dose**	**$IC50_{ratio,drug}$**	**CI**	**Type**	**$IC50_{ratio,drug}$**	**CI**	**Type**	**$IC50_{ratio,drug}$**	**CI**	**Type**	**$IC50_{ratio,drug}$**	**CI**	**Type**	**$IC50_{ratio,drug}$**	**CI**	**Type**
**5-Fluorouracil**	6h at 40.5°C	1,02	1,49	Antagonic	0,77	1,09	**Additive**	0,80	1,43	Antagonic	1,35	1,74	Antagonic	1,12	1,58	Antagonic
	6h at 41°C	1,06	1,58	Antagonic	1,11	1,47	Antagonic	1,05	1,76	Antagonic	1,66	2,1	Antagonic	1,09	1,62	Antagonic
	6h at 41.5°C	1,22	1,81	Antagonic	4,88	5,29	Antagonic	0,44	1,25	Antagonic	0,82	1,32	Antagonic	1,07	1,66	Antagonic
	12h at 40.5°C	0,29	0,83	**Synergic**	0,83	1,2	Antagonic	0,37	1,21	Antagonic	1,30	1,73	Antagonic	0,75	1,29	Antagonic
	12h at 41°C	0,20	0,82	**Synergic**	0,94	1,37	Antagonic	0,15	1,1	**Additive**	1,23	1,72	Antagonic	0,71	1,34	Antagonic
	12h at 41.5°C	0,24	0,93	**Additive**	0,39	0,87	**Synergic**	0,001	1,07	**Additive**	0,65	1,2	Antagonic	0,45	1,15	Antagonic
	24h at 40.5°C	0,18	1,05	**Additive**	0,18	0,96	**Additive**	0,07	0,94	**Additive**	0,33	1,04	**Additive**	0,80	1,58	Antagonic
	24h at 41°C	0,008	1,01	**Additive**	0,01	0,9	**Additive**	0,01	1,01	**Additive**	0,15	0,96	**Additive**	0,40	1,3	Antagonic
	24h at 41.5°C	2,95E-05	1,13	Antagonic	0,01	1,01	**Additive**	0,0007	1,13	Antagonic	0,03	0,95	**Additive**	0,006	1,02	**Additive**
**Gemcitabine**	6h at 40.5°C	1,44	1,9	Antagonic	1,96	2,28	Antagonic	0,88	1,5	Antagonic	1,18	1,57	Antagonic	1,07	1,53	Antagonic
	6h at 41°C	1,40	1,93	Antagonic	0,73	1,09	**Additive**	0,80	1,52	Antagonic	0,98	1,42	Antagonic	0,95	1,47	Antagonic
	6h at 41.5°C	1,30	1,88	Antagonic	-	-	-	0,42	1,22	Antagonic	0,89	1,39	Antagonic	1,15	1,74	Antagonic
	12h at 40.5°C	0,77	1,31	Antagonic	-	-	-	0,30	1,13	Antagonic	1,76	2,2	Antagonic	0,81	1,36	Antagonic
	12h at 41°C	0,30	0,91	**Additive**	0,70	1,13	Antagonic	0,13	1,09	**Additive**	1,78	2,27	Antagonic	0,74	1,37	Antagonic
	12h at 41.5°C	0,27	0,96	**Additive**	0,22	0,7	**Synergic**	0,0008	1,07	**Additive**	1,28	1,84	Antagonic	0,60	1,31	Antagonic
	24h at 40.5°C	0,17	1,05	**Additive**	0,01	0,79	**Synergic**	0,085	0,96	**Additive**	0,69	1,4	Antagonic	0,82	1,6	Antagonic
	24h at 41°C	0,026	1,03	**Additive**	0,002	0,89	**Synergic**	0,02	1,02	**Additive**	0,19	1,01	**Additive**	0,52	1,41	Antagonic
	24h at 41.5°C	0,001	1,13	Antagonic	-	-	-	0,003	1,13	**Additive**	0,04	0,95	**Additive**	0,007	1,02	**Additive**
**Cisplatin**	6h at 40.5°C	0,74	1,2	Antagonic	1,4	1,74	Antagonic	1,00	1,63	Antagonic	0,78	1,16	Antagonic	1,02	1,48	Antagonic
	6h at 41°C	0,73	1,26	Antagonic	1,48	1,85	Antagonic	1,04	1,76	Antagonic	0,72	1,16	Antagonic	1,01	1,54	Antagonic
	6h at 41.5°C	0,70	1,3	Antagonic	1,04	1,44	Antagonic	0,56	1,37	Antagonic	0,85	1,35	Antagonic	0,88	1,47	Antagonic
	12h at 40.5°C	0,43	0,97	**Additive**	0,77	1,14	Antagonic	0,75	1,58	Antagonic	0,75	1,18	Antagonic	0,93	1,48	Antagonic
	12h at 41°C	0,36	0,98	**Additive**	0,62	1,04	**Additive**	0,26	1,21	Antagonic	0,26	0,76	**Synergic**	0,76	1,39	Antagonic
	12h at 41.5°C	0,23	0,92	**Additive**	0,41	0,89	**Synergic**	0,007	1,08	**Additive**	0,007	0,56	**Synergic**	0,47	1,17	Antagonic
	24h at 40.5°C	0,08	0,96	**Additive**	0,08	0,86	**Synergic**	0,06	0,94	**Additive**	0,06	0,78	**Synergic**	0,49	1,27	Antagonic
	24h at 41°C	0,001	1	**Additive**	0,005	0,89	**Synergic**	0,02	1,02	**Additive**	0,02	0,84	**Synergic**	0,06	0,96	**Additive**
	24h at 41.5°C	8,50E-05	1,13	Antagonic	0,001	1	**Additive**	0,003	1,13	Antagonic	0,003	0,92	**Additive**	0,0001	1,01	**Additive**

Supplementary Figures

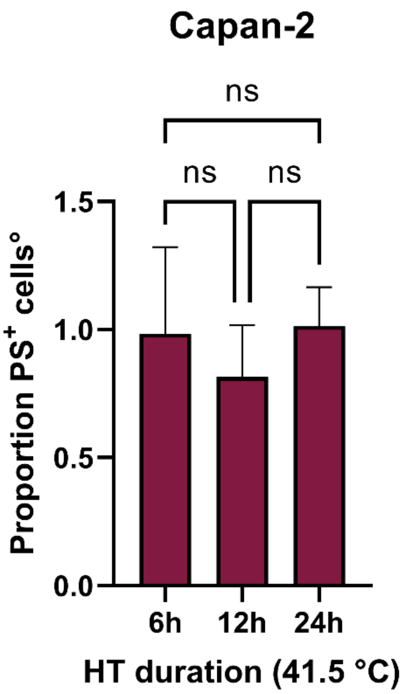

Supplementary Figure S1: Expression of phosphatidylserine (PS) on the outer membrane of Capan-2. No significant differences in PS positivity were observed.

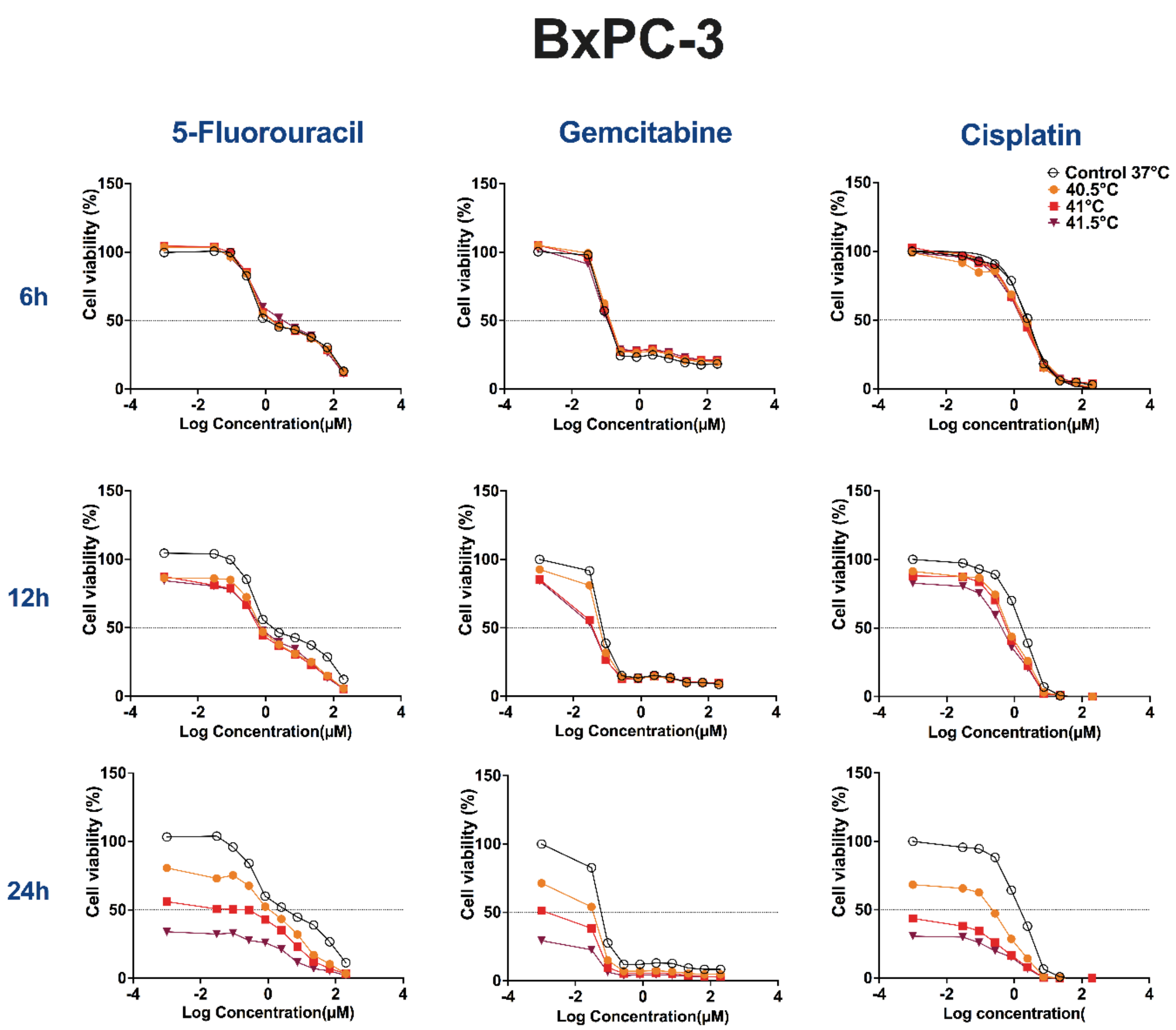

Supplementary Figure S2: Dose curve response of BxPC-3 to 5-fluorouracil, gemcitabine and cisplatin. These data represent triplicates.

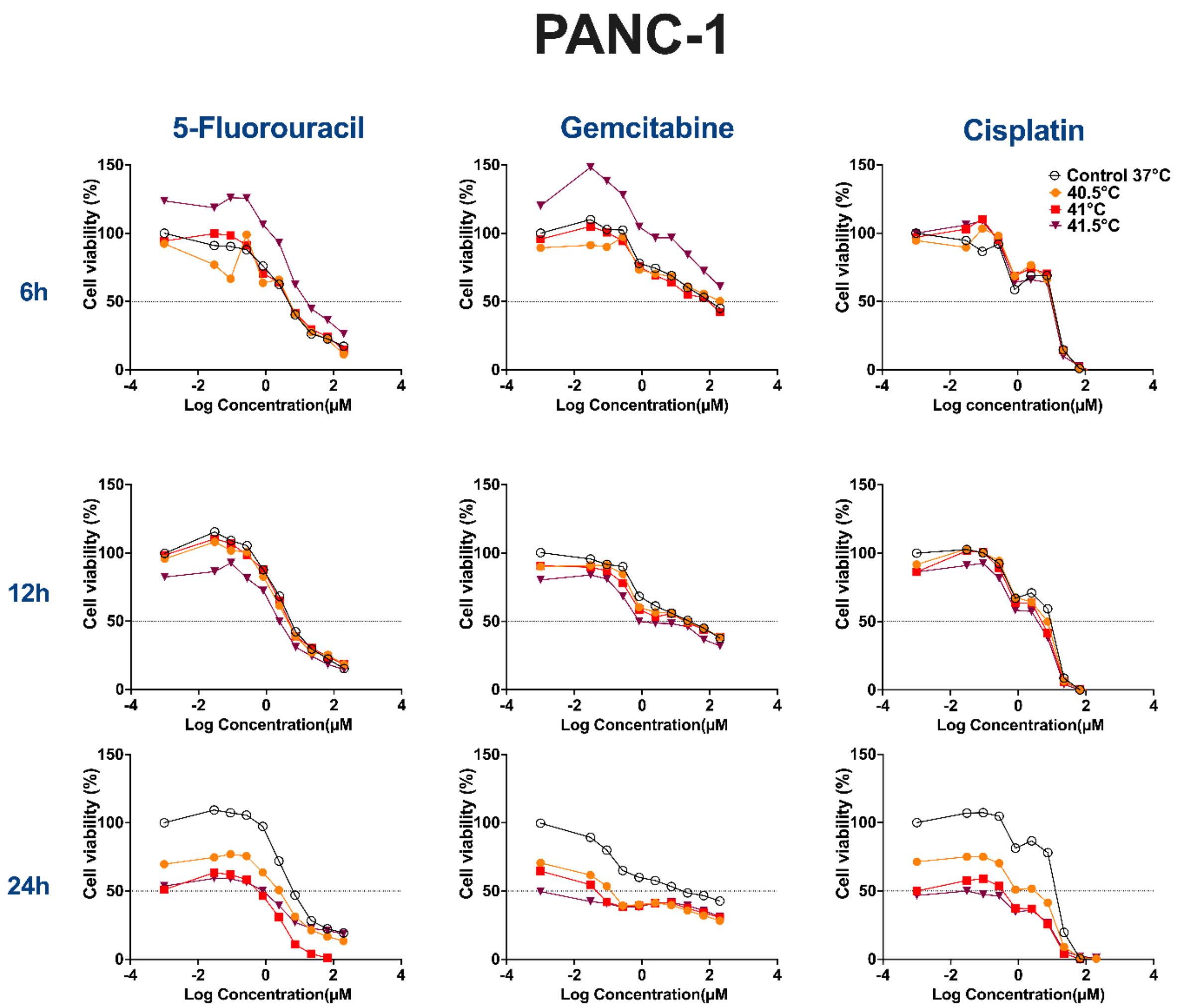

Supplementary Figure S3: Dose curve response of PANC-1 to 5-fluorouracil, gemcitabine and cisplatin. These data represent triplicates.

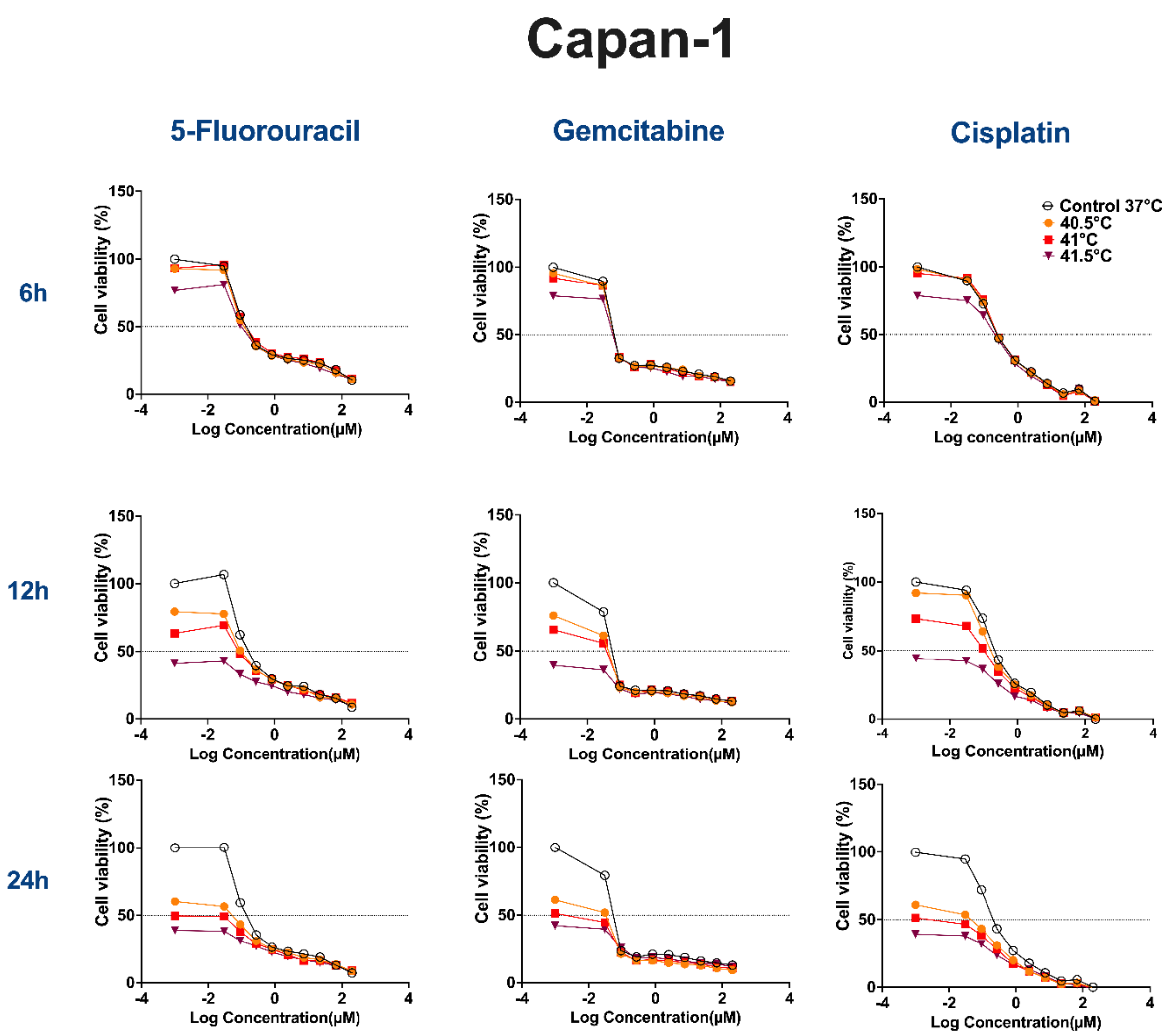

Supplementary Figure S4: Dose curve response of Capan-1 to 5-fluorouracil, gemcitabine and cisplatin. These data represent triplicates.

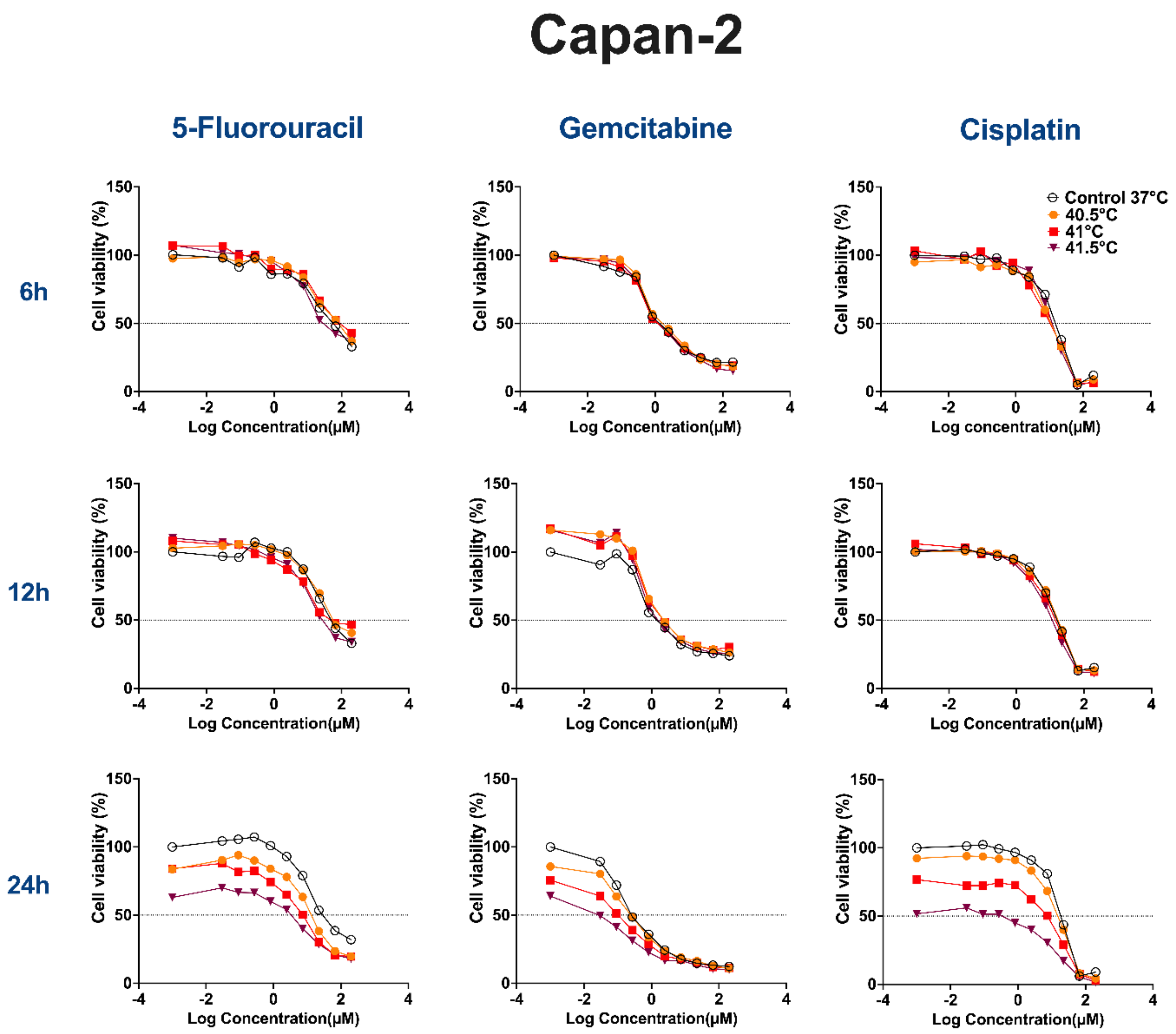

Supplementary Figure S5: Dose curve response of Capan-2 to 5-fluorouracil, gemcitabine and cisplatin. These data represent triplicates.

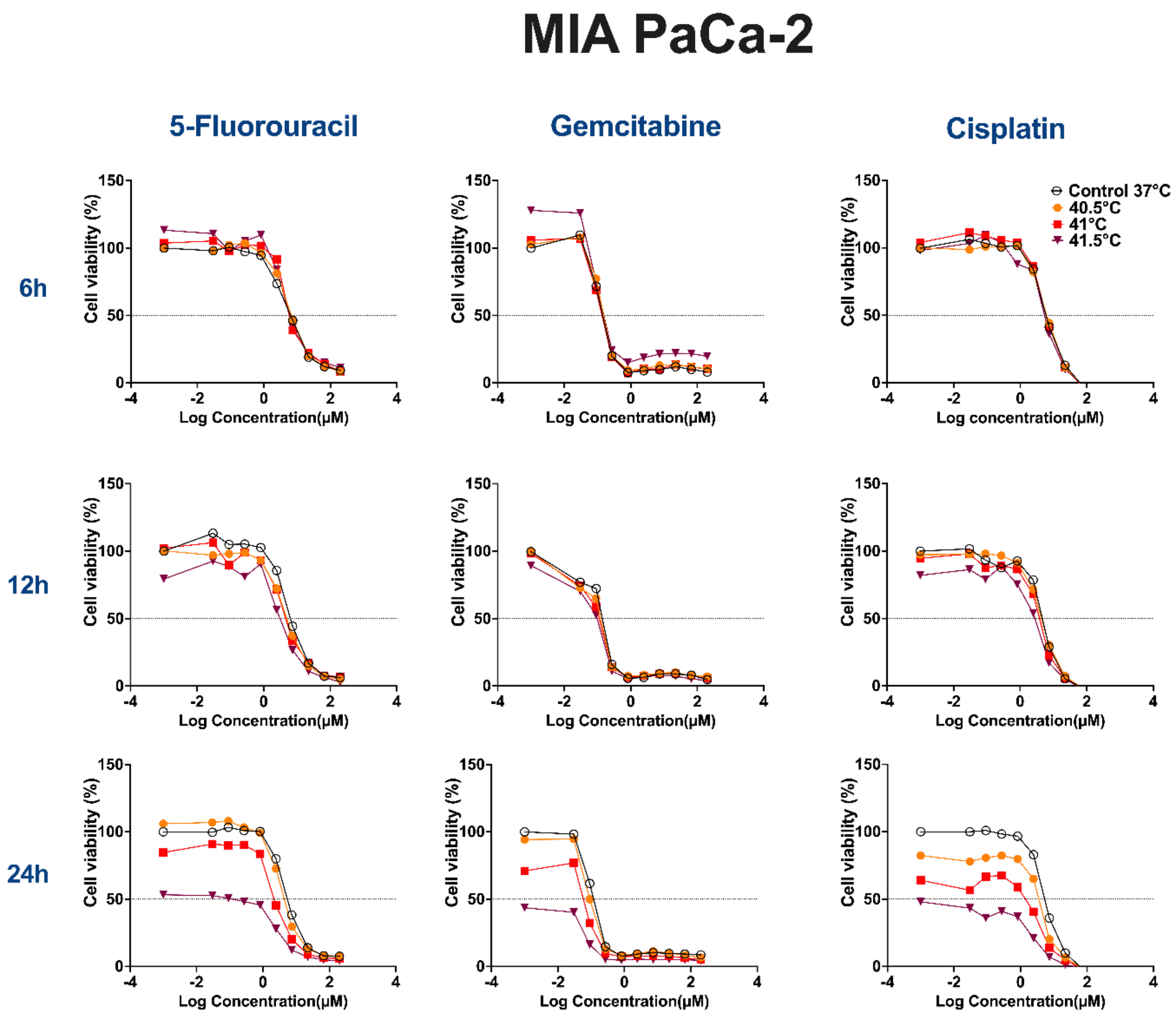

Supplementary Figure S6: Dose curve response of MIA PaCa-2 to 5-fluorouracil, gemcitabine and cisplatin. These data represent triplicates.
